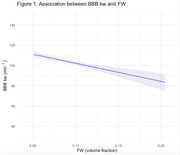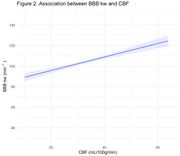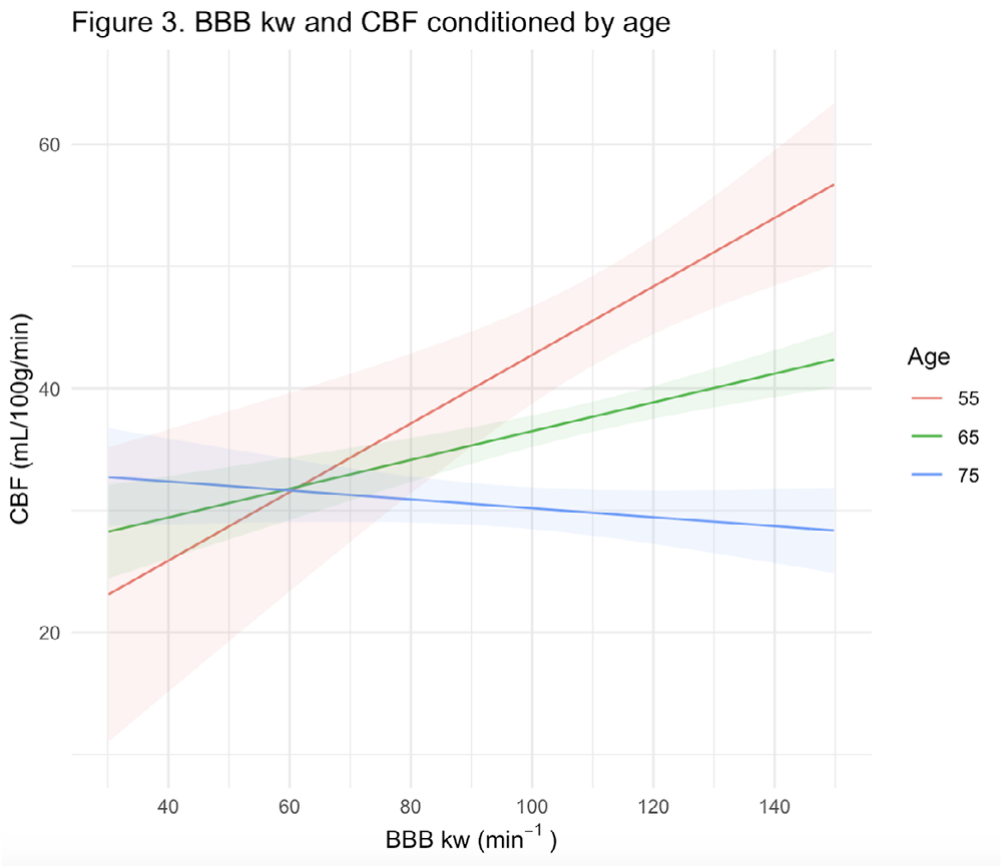# Investigation of blood‐brain barrier water exchange rate as a novel early indicator of neurovascular dysfunction and Alzheimer’s disease

**DOI:** 10.1002/alz.091069

**Published:** 2025-01-09

**Authors:** Ella Rowsthorn, Ming Ann Sim, William O'Brien, Stuart McDonald, Lucy Vivash, Terence J O'Brien, Meng Law, Trevor T.‐J. Chong, Xingfeng Shao, Danny JJ Wang, Matthew P. Pase, Ian H Harding

**Affiliations:** ^1^ Central Clinical School, Monash University, Melbourne, VIC Australia; ^2^ Turner Institute for Brain and Mental Health & School of Psychological Sciences, Monash University, Clayton, VIC Australia; ^3^ Alfred Health, Melbourne, VIC Australia; ^4^ University of Southern California, Los Angeles, CA USA; ^5^ QIMR Berghofer Medical Research Institute, Herston, QLD Australia

## Abstract

**Background:**

Growing evidence shows neurovascular dysfunction occurs early in Alzheimer’s disease (AD) and may be a useful early marker of pathology. Blood‐brain barrier water exchange rate (BBB kw) is a novel measure of fluid transport through the neurovascular unit. Lower BBB kw has been associated with vascular risk factors, but its relationship with age, vascular brain health and biomarkers of AD remains equivocal. The present study investigated the relationships between BBB kw, age, MRI measures of neurovascular integrity, and cerebrospinal fluid amyloid‐beta (Aβ).

**Method:**

The Brain and Cognitive Health (BACH) Cohort Study recruited 108 participants without dementia, aged 55‐80 (68.5% female; mean age=67 years) from the general community. Participants underwent a suite of MRI scans (3T Siemens Prisma) that allowed for quantification of BBB kw, enlarged perivascular space volume (ePVS), diffusion free water (FW), cerebral blood flow (CBF), and white matter hyperintensity volume (WMH). A subsample of 35 individuals also completed an optional lumbar puncture for Aβ_42_ and Aβ_40_ quantification. Linear regression models and linear mixed‐effects models were used to investigate the association between BBB kw and the outcomes. We also investigated whether there was an interaction effect of age on MRI measure relationships. All analyses were adjusted for age, sex and intracranial volume.

**Result:**

BBB kw was negatively associated with age (B(SE)=‐44.74(20.91), R^2^_partial_ =.04, p=.04), negatively associated with FW (B(SE)=‐87.86(29.03), R^2^_marginal_=.128, p=.006; Figure 1), and positively associated with CBF (B(SE)=0.02(0.08), R^2^_marginal_=.128, p=.018; Figure 2). BBB kw was not associated with ePVS or WMH (p>.05). The association between BBB kw and CBF was modified by age (R^2^_partial_=.06, p=.047), such that the relationship was stronger in younger participants (Figure 3). BBB kw was not associated with Aβ_42_/Aβ_40_ ratio (B(SE)=59.89(205.30), R^2^_partial_=<.01, p=.773) in the small subsample.

**Conclusion:**

Lower efficiency of fluid transport through the neurovascular unit was associated with markers of neurovascular health, including cerebral perfusion and fluid accumulation/white matter integrity. These findings suggest BBB kw as a useful imaging tool for studying neurovascular unit integrity in the context of brain aging. Associations of BBB kw across the continuum of AD requires further investigation.